# Autoantibody-Negative Immune-Mediated Necrotizing Myopathy Responds to Early and Aggressive Treatment: A Case Report

**DOI:** 10.7759/cureus.7827

**Published:** 2020-04-25

**Authors:** Dario A Marotta, Adena Zadourian, Maxwell J Jabaay, Ali Kesserwani, Hassan Kesserwani

**Affiliations:** 1 Department of Research, Alabama College of Osteopathic Medicine, Dothan, USA; 2 Department of Neurology, Division of Neuropsychology, University of Alabama, Birmingham, USA; 3 Neurology, Dothan Neurology, Dothan, USA; 4 Neurology, Flowers Medical Group, Dothan, USA

**Keywords:** immune-mediated necrotizing myopathy, autoantibodies, dual immunotherapy, anti-hmgcr, anti-srp

## Abstract

Immune-mediated necrotizing myopathy (IMNM) is a rare idiopathic disease that is further classified by the presence of serum antibodies. A modicum of patients lack serum autoantibodies. Significantly elevated creatine kinase (CK) is highly characteristic of IMNM. The pathophysiology of IMNM is partially understood, and effective treatment options are limited, particularly in patients without serum autoantibodies. In this case, we report a 76-year-old male presenting with a four-month history of proximal muscle weakness. Muscle biopsy and serology confirmed the diagnosis of autoantibody-negative IMNM. Early and aggressive treatment with high-dose steroids and a course of intravenous immunoglobulin significantly reduced the patient’s symptoms and CK within three months. This case serves as an example of an effective treatment outcome in a patient with this rare idiopathic necrotizing myopathy.

## Introduction

Immune-mediated necrotizing myopathy (IMNM) is a rare debilitating idiopathic myopathy. Although the exact prevalence is under debate, contemporary literature suggests IMNM affects about 7-11 in 100,000 people yearly in the United States [1-3]. Patients with IMNM have been classified by the presence or absence of serum autoantibodies, such as anti-signal recognition particle (SRP), anti-hydroxy-3-methylglutaryl-CoA reductase (HMGCR), and autoantibody-negative. Each subset of IMNM is believed to have its own unique characteristics, pathogenesis, and potential treatment strategy [4]. The majority of IMNM patients possess either anti-SRP or anti-HMGCR antibodies, while only 10% of patients with IMNM are autoantibody-negative [2]. The literature lacks randomized controlled trials for these patients, which makes treatment reliant on anecdotal and case series reports [5]. Here we report a 76-year-old male with biopsy and serology confirmed autoantibody-negative IMNM who responded well to early and aggressive treatment with high-dose steroids and a course of intravenous immunoglobulin (IVIG). This treatment significantly reduced the patient’s symptoms allowing him to return to his usual activities of daily living.

## Case presentation

A 76-year-old Caucasian male presented to the clinic with a four-month history of progressive and proximal bilateral muscle weakness causing significant difficulty climbing into his vehicle, rising from a chair, ambulating, and lifting heavy items. He was no longer able to drive a school bus. His past medical history was significant for hypertension, hyperlipidemia, cerebrovascular disease, and urinary retention. The patient’s medications included aspirin 81 mg daily, amlodipine 5 mg daily, hydrochlorothiazide 25 mg daily, and tamsulosin 0.4 mg daily. The patient had a prior three-year history of atorvastatin use, which was discontinued three years prior to presentation due to diffuse myopathic symptoms. His hyperlipidemia had been managed by diet and exercise alone since statin discontinuation. The patient’s family history was significant for unspecified malignancy in his mother, father, and sister. His social history was significant for a 25 pack-year smoking history. 

Physical examination revealed a waddling gait and the inability to stand from a seated position with his arms folded. There was no evidence of bulbar involvement, ulnar wasting of the forearms, or difficulty standing on the heels. There was no evidence of heliotrope rash on the face, or hyperkeratotic erythema on the hands, knuckles, or fingers. Muscle strength testing with the Medical Research Council grading scale revealed weakness in bilateral shoulder abduction (4/5) and bilateral hip flexion (4/5). Upper and lower extremity reflexes were normal throughout (2/4). 

Electromyography (EMG) of the right upper extremity (proximal and distal), left upper extremity (proximal and distal), and cervical paraspinal muscles revealed florid and widespread fibrillation potentials and positive sharp waves. Myopathic units, with early and full recruitment, were present in proximal muscle groups. Nerve conduction studies of the right upper extremity and bilateral lower extremities showed mild axonal sensorimotor polyneuropathy. Initial serology revealed elevated total CK of 13,510 units/L (normal 24-204). Acetylcholinesterase-binding antibodies and anti-striation antibodies were negative. A comprehensive paraneoplastic panel was negative.

**Figure 1:**
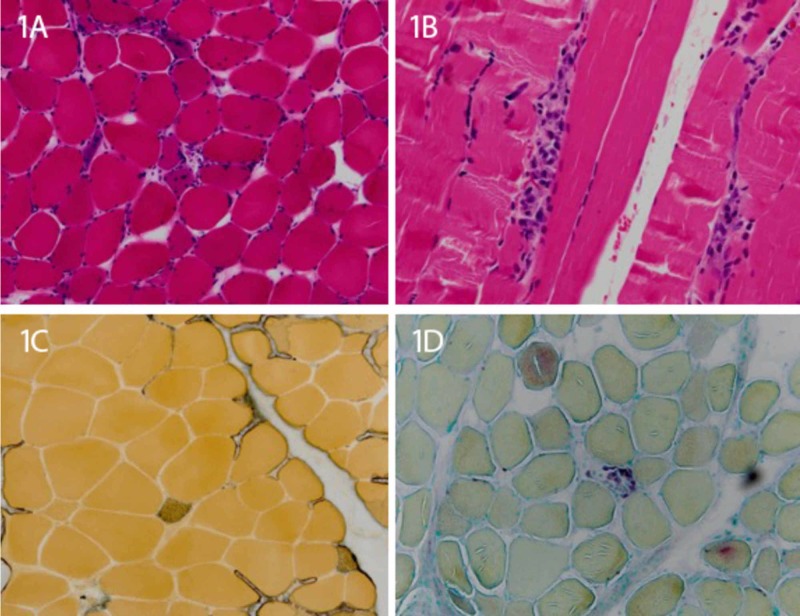
Left quadricep muscle biopsy consistent with immune-mediated necrotizing myopathy 1A and 1B: Hematoxylin and eosin stain with moderation in muscle fiber size, scattered necrotic fibers, and minimal-to-no lymphocytic inflammation; 1C: Acid phosphatase stain shows few degenerating fibers; 1D: Alkaline phosphatase stain shows several regenerating fibers.

The patient was started on intravenous solumedrol 500 mg weekly for four weeks. The patient’s muscle weakness progressed and a left quadricep biopsy was collected. As shown in Figure 1, the biopsy revealed moderate variation in muscle fiber size, scattered necrotic fibers, and minimal-to-no lymphocytic inflammation, consistent with immune-mediated necrotizing myopathy (IMNM). Serology testing revealed the patient did not possess anti-SRP or anti-HMGCR antibodies. The patient’s condition continued to decline despite solumedrol treatment. The patient was admitted to the hospital and administered dual immunomodulation therapy consisting of oral prednisone 60 mg daily and intravenous immunoglobulin 0.4 g/kg body weight daily for five days. The patient’s myalgias slightly improved and he was discharged with prednisone 60 mg daily until the next follow-up appointment.

Fourteen days later, the patient’s CK levels significantly decreased to a level of 2,500 U/L. The patient remained on high-dose prednisone monotherapy and serial blood draw revealed CK levels continued to decline to 820 U/L after about 90 days. The patient’s symptoms nearly resolved enough to allow him to fully reengage with activities of daily living; he became independent with ambulation and was able to return to driving a bus.

## Discussion

IMNM is a rare and serious disease that can have a drastic impact on a patient’s quality of life if not quickly managed [6]. Muscle biopsy remains the mainstay of diagnosis. In contrast with polymyositis, dermatomyositis, and inclusion body myositis, IMNM biopsies contain overtly necrotic muscle fibers with minimal or absent inflammation [5-7]. Following a muscle biopsy, serum analysis for autoantibodies can help guide treatment using evidence-based practice. However, in the case of autoantibody-negative IMNM, the literature surrounding treatment is lacking. This case report highlights the importance of documented successful treatment outcomes in these patients. In this case, we report a 76-year-old male with autoantibody-negative IMNM who responded to continuous high-dose prednisone with a loading dose of IVIG.

As it stands, IMNM is composed of three defined sub-categories based on the presence of serum autoantibodies: 1) anti-HMGCR; 2) anti-SRP; 3) autoantibody-negative [6,8]. Together, anti-HGMCR and anti-SRP represent the majority of IMNM cases. HMGCR is the rate-limiting enzyme involved in cholesterol synthesis and is frequently associated with prior statin use [9]. SRP is a ribonucleoprotein involved with protein localization to the rough endoplasmic reticulum. Autoantibodies against SRP often occur at a younger age, have more severe muscle weakness, and have been shown to respond to rituximab [10]. Conversely, autoantibodies against HMGCR commonly present in adulthood and have been successfully treated with IVIG monotherapy [11]. Autoantibody-negative IMNM is less understood and minimal diagnostic and treatment guidance is available in the literature.

Patients with IMNM often present with symmetric bilateral proximal muscle weakness which can occur acutely, in a matter of weeks, or slower, over the course of several months [5,7]. Gross elevation in serum CK is highly characteristic of IMNM [2,7]. CK elevations can be used for disease prognostication in that they correspond with increased disease severity and necrosis [2,5]. Median CK values in IMNM are between 4,000-7,000 U/L [7,12]. In this case, the patient’s initial CK level was 13,510 U/L. CK levels and the findings on muscle biopsy effectively differentiated this patient’s disease process from other inflammatory myopathies, such as dermatomyositis, which have a much lower median CK value of approximately 700 U/L [2].

Several risk factors have been associated with IMNM such as statin exposure, malignancy, and viral infections, two of which stand out as attributable risks in this patient [2,7,12]. First, the patient had a prior history of statin-induced myopathy following three years of atorvastatin use. The medication was discontinued three years prior to the onset of IMNM and reportedly resolved the myopathy within a matter of days. The patient’s history of statin use, in combination with his anti-HMGCR seronegative status, suggests there may be an overlap in pathogenesis between anti-HMGCR and autoantibody-negative IMNM. Secondly, the patient had a significant family history for malignancy in his mother, father, and sister. At the time of presentation, the patient did not display signs or symptoms consistent with malignancy. A comprehensive paraneoplastic panel and CT of the chest, abdomen, and pelvis were negative. However, it is well documented that IMNM, particularly autoantibody-negative IMNM, has the highest risk of concomitant neoplastic disease [8,12].

Currently, there are no documented randomized clinical drug trials pointing to an effective treatment for IMNM. High-dose steroids, IVIG, and immunosuppressive agents such as methotrexate, azathioprine, and rituximab are generally considered acceptable treatment options for IMNM [2,13-14]. Early and aggressive immunotherapy with at least two agents has improved the prognosis for some patients [2,6-7,15]. However, those who are positive for either anti-SRP or anti-HMGCR have been shown to have mixed results with steroid monotherapy [7,16]. Even with treatment, up to half of IMNM patients experience continued muscle weakness after two years [4].

A unique treatment challenge exists with IMNM patients. By the time a patient initially presents to the clinic, obtains a confirmatory biopsy, and subsequent antibody serology, a considerable amount of time can elapse. In this case, we immediately initiated high steroid treatment prior to IMNM biopsy confirmation. When the patient continued to deteriorate, we admitted the patient to the hospital for IVIG administration (0.4 g/kg/d for five days), at which time the patient’s symptoms began to plateau. Continued high dose steroid therapy for a period of 90 days led to a near-complete resolution of symptoms. This strategy reduced the patient’s CK levels from 13,510 U/L at the time of presentation down to 820 U/L, three months later. Ultimately, the patient regained activities of normal living and will remain under observation. This case supports the use of early and aggressive administration of high dose steroids with a pulse dose of IVIG in patients with autoantibody-negative IMNM.

## Conclusions

Autoantibody-negative IMNM presents unique challenges to treatment. Here we report a case of early and aggressive administration of high-dose steroids with a pulse dose of intravenous immunoglobulin in a 76-year-old male, leading to near-complete resolution of symptoms and a dramatic reduction in CK.
